# Supplementary material for: On the fiscal deficit and economic growth in sub-Saharan Africa: A new evidence from system GMM
Source: PLoS One. 2024 Jul 22;19(7):e0303825. doi: 10.1371/journal.pone.0303825 (PMC11262640; doi:10.1371/journal.pone.0303825)
Supplement: Attachment — Submitted filename: Comments 1.pdf [file pone.0303825.s003.pdf]

In my earlier comments, I raised some concerns to enhance the quality of the paper so as to merit publication in Plos One. However, upon reviewing the current version of the manuscript, I have noticed that the concerns I raised previously have not been addressed in the revised manuscript.

To clarify, I have listed my previous concerns (1-6) below:

1. The write-up needs proofreading. In its current state, the paper is difficult to read and appreciate its contributions.
2. Originality - Authors stated (see page 3) that one of their contributions to the debate is the methodology employed but they failed to clearly state the weakness in the PMG methodology (used by Awolaja & Esefo, 2020) which GMM is addressing or why GMM results are more reliable/superior to PMG
3. The empirical literature review was poorly carried out as it generally reported the findings of previous studies without any form of discussion.
4. The authors transformed all variables to their natural logarithm including fiscal deficit. Meanwhile, authors computed fiscal deficit as the difference between tax revenue (% of GDP) and government final consumption expenditure (% of GDP) which suggests that there would be a lot of negative values yet authors proceeded to take the natural logarithm of these values (which is undefined) without any additional information

5. Other errors detected in the manuscript have been listed below:

- Authors interpret some I(0) variables as I(1) in Table 3 (page 16)
- On page 16 authors wrote **“We are now confident enough that the result of our two-step system GMM is free from the risk of non-spurious regression.”** but it’s not clear whether the authors meant spurious regression instead of non-spurious regression
- On page 18, the authors wrote **“In addition to this, coefficient of real interest rate (logRIR) is found to have positive coefficient implying that a one percent changes in rate of real interest is associated with a 0.074 percent decline in economic growth, other things remaining constant.”**, which is incorrect.
- On the same page (i.e. page 18), the authors stated **“Thus, the result of the study is consistent across all estimated models.”** but the real effective exchange rate and inflation variables have opposite signs in model 3 compared to model 1 and 2 (see Table 5).
- On page 18: **“The result of the two-step system GMM revealed that a percentage change in the rate of inflation (consumer price index), all other things kept constant, is associated with a -0.035 decline in economic growth of SSA during 2011-2021. This result remained consistent across all the three columns of table 5.”** but inflation has a positive sign in column 3.

6. Overall, the paper has not convincingly demonstrated the relevance of its contribution to the fiscal deficit and growth debate. Apart from the errors listed above, there are also too many typos, grammatical errors, and poor sentence constructions which may reduce the interest of the reader. The paper needs to be significantly improved to merit publication in PLOS ONE.

Although the authors may have attempted to proofread the manuscript, there is still room for improvement in terms of language quality to enhance clarity and reader engagement. It is worth noting that the authors have not addressed the remaining issues (numbered 2-5) and have also failed to provide any rebuttals. Some of these issues are crucial in understanding the paper's contribution and evaluating the accuracy and dependability of the findings.
