# Supplementary material for: On the fiscal deficit and economic growth in sub-Saharan Africa: A new evidence from system GMM
Source: PLoS One. 2024 Jul 22;19(7):e0303825. doi: 10.1371/journal.pone.0303825 (PMC11262640; doi:10.1371/journal.pone.0303825)
Supplement: Attachment — Submitted filename: PLOS ONE - Review.docx [file pone.0303825.s001.docx]

PLOS ONE - Review

**Article Title: Effects of Fiscal deficit on economic growth in Sub-Saharan Africa: Fresh Evidence from System GMM.**

The main objective of the study is to examine the pathways of fiscal deficit and economic growth of 42 sub-Saharan Africa countries using two-step system GMM for the period of 2011-2021. The main results from the study is that fiscal deficit have a positive effect on economic growth of the countries understudy.

**Comments:**

1. I have some reservations with the topic. Why not “Effects of fiscal deficit on economic growth in sub-Sahara Africa: A **new** evidence from system GMM”. I am not comfortable with the word “fresh”.
2. The effects of fiscal deficit on economic growth is overly researched even before the advent of the COVID-19 pandemic so what different or novelty is the study bringing on board. In fact, during the financial crisis in 2008 fiscal deficit became the issue in town. The study’s assertion in page 3 paragraph 2 that literature on impacts of fiscal deficits and economic growth is limited is not true and it defeats the discussions from the introductory page to that paragraph because the introductory discussions mainly focused on works that have been done on the subject. So why that assertion?
3. The rational and the motivation need to explicitly stated. The study’s rational and motivation needs to be re-enforced because the topic is overly researched in the macroeconomic literature. If it is about the updated data and new methodology of system GMM which is not new as claimed by the author(s) that needs to be emphasized in the study.
4. Under section 3.1, the author mentioned that the study followed the endogenous growth model theory but that model was not discussed under the theoretical review.
5. Under Table 2 caption, “sample” should be “Sample”.
6. Under section 3.2, the discussions on endogenous growth model should be done under the theoretical review and the author(s) should make references to it when specifying the model under section 3.2.
7. To be fair, the methodology used is appropriate and the pre-estimation test were adequately provided. However, the model specification is problematic. The study specifies a growth model and as such, should include other factors/variables that are known to impact economic growth and not only those chosen. As it stands, the model is misspecified. In recent times, literature on economic growth have found institutional and governance variables to impact economic growth and I expect the author(s) to include some of them.
8. The author(s) needs to provide substantiated reasons for the findings. It is not just presenting what is in the tables but adding economic explanations for these findings.

**General Comments**

1. The study needs a proof reader to correct some grammatical errors in the work. There are so many subject-verb agreement errors and inconsistencies in the some of the sentences.
2. The majority of issues from this study has to do with its conceptualization that needs to be addressed before the paper can be published else the paper does not add anything to the literature.
