# Supplementary material for: On the fiscal deficit and economic growth in sub-Saharan Africa: A new evidence from system GMM
Source: PLoS One. 2024 Jul 22;19(7):e0303825. doi: 10.1371/journal.pone.0303825 (PMC11262640; doi:10.1371/journal.pone.0303825)
Supplement: Attachment — Submitted filename: Article 3 PLos response reveiwers comment.docx [file pone.0303825.s002.docx]

Date: 9^th^ September, 2023

**Response to Reviewers’ Comments**

**Manuscript No.:** PONE-D-23-23990

**Title:** Effects of Fiscal Deficit on Economic Growth in Sub-Saharan Africa: A New Evidence from System GMM

**Journal:** PLOS ONE

**Dear Reviewers,**

Greetings of the day!

We are thankful to the reviewers for taking the time to assess our manuscript, for their careful reading, and for their suggestions and valuable comments which helped us to substantially improve the quality of our paper. In revising the manuscript, we have carefully considered all the raised comments and suggestions. We have attempted to succinctly explain the changes made in reaction to all comments. Our reply to each comment in a point-by-point fashion is given in what follows

1. **Regarding your concerns about the topic of the article**

Respected reviewers, thank you for your comment regarding the topic of our study. We have accepted it positively. In line with your comments, we have now changed the topic by avoiding the word Fresh. The corrected topic of the study is now:

*Effects of Fiscal Deficit on Economic Growth in Sub-Saharan Africa: A New Evidence from System GMM*

2. **On your concerns regarding introductory pages**

Dear Reviewers, thank you so much for your constructive comments regarding the introduction of our work.

We have now corrected and articulated the introduction in a more articulated way. For instance, we have now solved the issue you raised regarding page 3 paragraph 2 of the original manuscript.

Further, the paragraph that shows the organization of the study is included at the end of the introduction section.

3. **Regarding your concerns about the rationale and the motivation of the study**

Dear reviewers, we would also like to thank you for your comment that improved our paper.

The rationale and the motivation of the study are now explicitly explained (paragraphs 3 and 4 on page 3 of this manuscript).

4. **Regarding your concerns about the Literature Review**

In line with your comments regarding the endogenous growth model in the theoretical literature review (see highlighted paragraphs on page 5 before section 2.2).

5. **On your concerns:** Under Table 2 caption, “sample” should be “Sample”

Thank you very much, We have corrected editions and made a proof proofreads to substantially improve the paper.

6. **On Your concerns in section 3.2 of the manuscript**

Following your comments, we have made references to references to it when specifying the model under section 3.2. We have improved by making the connection between the literature section and the methodology section as per your comments. Thank you so much for your constructive comments.

7. **Regarding your concerns about model specification**

Dear reviewers, we would like to thank you for your previous comments regarding the model. You have said:

*‘To be fair, the methodology used is appropriate and the pre-estimation test was adequately provided. ’* We are happy to hear this from you.

We have substantially improved the model specification section in line with your comments.

The main objective of our study is to examine the long and short-run effects of fiscal deficit in SSA countries. Thus, we have a fiscal deficit as a variable of interest. The rest are control variables. Our control variables included in the study are inflation (INF), real effective exchange rate (RER), real interest rate (IR), and gross fixed capital formation (GFCF). These variables are the fundamental problems of SSA countries. They are the major determinants of growth in Africa. They have direct impacts on growth. This is why we included them in our model.

Since our objective is not to look at how governance and institutional qualities affect the linkage between fiscal deficit and economic growth, we haven’t included them in our study for now. This may be dubbed as the limitation of this study. Regardless of this, SSA countries’ policymakers want to know the short-run and long-run effects of fiscal deficit on economic growth. This is because growth is mainly financed by deficit and foreign borrowing.

8. **On your comments regarding discussions and findings**

Dear reviewers, thank you so much for your comments once again. Frankly speaking, we have learned a lot and our paper has got a major benefit from your comments.

Following your comments, we have interpreted our results substantially. We have provided reasons. We have included the result's economic meaning. It’s beyond mere interpretation of results from the table (We highlighted section 4 in red on pages 15 to 24).

**On your general Comments**

1. **On your suggestion regarding proofreading the manuscript**

Following your comments, we have re-read our manuscripts and corrected grammatical errors in our work. We have also eliminated subject-verb agreement errors and inconsistencies in some sentences in our manuscript. Generally, we have seriously worked on this.

2. **Regarding your concerns about the conceptualization of the study**

Dear reviewers, we have accepted your comments strongly and positively.

The major concern of our study is to examine the long-run and short-run effects of fiscal deficits in SSA. Our study generates new evidence regarding this for the last ten years (second decades of the 21^st^ century). Fiscal deficit was a major concern of literature during the global financial crises. Now, the world experienced a new event, a global pandemic that boosted fiscal deficit and inflation. It deteriorated foreign exchange and real interest rates. It seriously harassed global capital formation. Due to this, SSA countries are currently in a dilemma on whether to finance their growth using fiscal deficit or not. Thus, the examination of the long and short-run effects of fiscal deficit has magnificent importance for policymakers.

Moreover, please look at empirical literature in SSA countries. You can check the online. They are very limited. Contrary to this, look at the budget deficit of SSA countries. It is rising. The existing literature are reporting contradicting findings. Thus, Policymakers are in confusion. Thus, our current study has a vital contribution to the literature on SSA in this area. For that matter fiscal deficit is the fundamental macroeconomic problem of developing countries. As it adds a drop to the limited existing literature, we strongly believe that our current study has a significant contribution to the literature.

On top of that in developing countries where government is more powerful, fiscal policy is the main tool of economic stabilization. A fiscal deficit is just the difference between government expenditure and government revenue. SSA countries are very much concerned about the difference in their income and expenditure. Thus, studying fiscal deficit is important for SSA literature.

Finally, we would like to thank once again all the reviewers for taking the time to review our manuscript, for their relevant remarks and comments, and especially for their specifications which helped us to improve the quality of our paper.

Sincerely yours, Isubalew Daba Ayana
