# Supplementary material for: On the fiscal deficit and economic growth in sub-Saharan Africa: A new evidence from system GMM
Source: PLoS One. 2024 Jul 22;19(7):e0303825. doi: 10.1371/journal.pone.0303825 (PMC11262640; doi:10.1371/journal.pone.0303825)
Supplement: Attachment — Submitted filename: Article 3 PLos R2 response to reveiwers.docx [file pone.0303825.s004.docx]

Date: 14^th^ November, 2023

**Response to Reviewers’ Comments**

**Manuscript No.:** PONE-D-23-23990R1

**Title:** Effects of Fiscal Deficit on Economic Growth in Sub-Saharan Africa: A New Evidence from System GMM

**Journal:** PLOS ONE

**Dear Reviewers,**

Greetings of the day!

We are thankful to the reviewers for taking the time to assess our manuscript, for their careful reading, and for their suggestions and valuable comments which helped us to substantially improve the quality of our paper. In revising the manuscript for the second time, we have carefully considered all the raised comments and suggestions. We have attempted to succinctly explain the changes made in reaction to all comments. Our reply to each comment in a point-by-point fashion is given in what follows

1. **On comment 1:** *The write-up needs proofreading. In its current state, the paper is difficult to read, and appreciate its contributions.*

**Answers:**

Dear reviewers, thank you so much for your comments. We have accepted your comments strongly and positively. Following your comments, we have re-read our manuscripts to enhance clarity and reader engagement. We have eliminated too many typos, grammatical errors, and poor sentence constructions. We believe that your comments made our manuscript smarter than before.

2. **On comment 2:** *Originality - Authors stated (see page 3) that one of their contributions to the debate is the methodology employed but they failed to clearly state the weakness in the PMG methodology (used by Awolaja & Esefo, 2020) which GMM is addressing or why GMM results are more reliable/superior to PMG*

**Answer:**

Dear reviewers, we have accepted your comments and suggestions positively. We have now explained the major reason why system GMM is superior to PMG. ( page 3 last paragraph), the highlighted paragraph. Your constructive comments are well appreciated.

3. **On comment 3:** *The empirical literature review was poorly carried out as it generally reported the findings of previous studies without any form of discussion.*

**Answer:**

Dear reviewers, we are very grateful for your comments on the literature section of our manuscript. Following your good comments, we have written the literature section more attractively. We have linked the ideas. We have included a detailed discussion regarding the findings. It is now more beautiful with a comprehensive discussion. The literature portion is now enriched. For convenience, The entire changes made in the literature review section are highlighted. Thank you for your constructive comments.

4. **On comment 4:**

*The authors transformed all variables to their natural logarithm including fiscal deficit. Meanwhile, authors computed fiscal deficit as the difference between tax revenue (% of GDP) and government final consumption expenditure (% of GDP) which suggests that there would be a lot of negative values yet authors proceeded to take the natural logarithm of these values (which is undefined) without any additional information*

**Answer**

Dear reviewers thank you once again for raising another essential question that improved our manuscript. Sure, the fiscal deficit variable is the difference between government revenues and government expenditures. We have faced some negative values. It makes no sense when we convert the negative value to a natural log. However, we have handled this problem scientifically. Page 15 above section 3.3 has a solution for this. It is written like this. Before transforming the value of a major explanatory variable, fiscal deficit, we faced some negative values of fiscal deficit as a percentage of GDP when we calculated the difference between government revenue and government expenditure. Since the log of the negative number is none in economics, as it is in mathematics, we used the most common technique of transforming to handle the negative values in our fiscal deficit data set. To do this, we added a constant value to our fiscal deficit data set before applying the log transform. This method is preferred as it is popular, easy, and more appropriate for managing a few negative numbers that appear in the data. Now, it is meaningful. Thank you for your comment that made our manuscript smarter.

5. **On Comment 5:** *Other errors detected in the manuscript have been listed below: • Authors interpret some I(0) variables as I(1) in Table 3 (page 16) • On page 16 authors wrote “We are now confident enough that the result of our two-step system GMM is free from the risk of non-spurious regression.” but it’s not clear whether the authors meant spurious regression instead of non-spurious regression • On page 18, the authors wrote “In addition to this, coefficient of real interest rate (logRIR) is found to have positive coefficient implying that a one percent changes in rate of real interest is associated with a 0.074 percent decline in economic growth, other things remaining constant.”, which is incorrect. • On the same page (i.e. page 18), the authors stated “Thus, the result of the study is consistent across all estimated models.” but the real effective exchange rate and inflation variables have opposite signs in model 3 compared to model 1 and 2 (see Table 5). • On page 18: “The result of the two-step system GMM revealed that a percentage change in the rate of inflation (consumer price index), all other things kept constant, is associated with a -0.035 decline in economic growth of SSA during 2011-2021. This result remained consistent across all the three columns of table 5.” but inflation has a positive sign in column 3.*

**Answer:**

Dear reviewers, we would like to thank you for your time again. You have given a lot of detailed comments under comments 5. Many are related to Table 3 and Table 5. We have looked carefully into them and corrected all of them. We have highlighted them carefully. In Table 3( page 19 of the revised manuscript), we carried out stationarity at the level and first difference. The result shows that all variables are stationary at first difference. Some variables are not stationary at the level. For system GMM, we need data to be stationary. It might be either at level or first difference. Variables that are not stationary at the level are stationary at the first difference. The corresponding decisions were provided at both I(0) and I(1) in both unit root test cases. Thus, the major concern here is solved.

Finally, we would like to thank once again all the reviewers for taking the time to review our manuscript, for their relevant remarks and comments, and especially for their specifications which helped us to improve the quality of our paper.

Sincerely yours, Isubalew Daba Ayana
