# Supplementary material for: On the fiscal deficit and economic growth in sub-Saharan Africa: A new evidence from system GMM
Source: PLoS One. 2024 Jul 22;19(7):e0303825. doi: 10.1371/journal.pone.0303825 (PMC11262640; doi:10.1371/journal.pone.0303825)
Supplement: Attachment — Submitted filename: PLOS ONE - Review_2.docx [file pone.0303825.s005.docx]

**General Comments**

To be fair, the author(s) need to address the following issues:

1. The rationale for the study is not captivating. Just like I said before the author(s) need to still motivate the work.
2. The grammatical errors in the paper need to be looked at. There are so many subject-verb agreement errors and inconsistencies in some of the sentences. I advised earlier for the service of a proofreader and I still think the author(s) need that.
