# Supplementary material for: On the fiscal deficit and economic growth in sub-Saharan Africa: A new evidence from system GMM
Source: PLoS One. 2024 Jul 22;19(7):e0303825. doi: 10.1371/journal.pone.0303825 (PMC11262640; doi:10.1371/journal.pone.0303825)
Supplement: Attachment — Submitted filename: Article 3 PLos R3 response to reveiwers.docx [file pone.0303825.s006.docx]

Date: 2^nd^ March, 2024

**Response to Reviewers’ Comments**

**Manuscript No.:** PONE-D-23-23990R2

**Title: On the Fiscal Deficit and Economic Growth in Sub-Saharan Africa: A New Evidence from System GMM**

**Journal:** PLOS ONE

Greetings, Reviewers
Salutations for the day!
We appreciate the reviewers' time in evaluating our article, their attentive reading, and their insightful recommendations and remarks, which enabled us to significantly raise the caliber of our work. The second revision of the article has taken into account all of the pointed criticisms and recommendations. We have tried to provide a clear explanation of the modifications made in response to all feedback. What follows is a point-by-point summary of our response to each comment.

1. **On comment 1:** 1. The rationale for the study is not captivating. Just like I said before the author(s) need to still motivate the work.

**Answers:**

Greetings, reviewers Your thoughts and opinions have been positively received by us. We appreciate your feedback, which made our job better. We have now stated the study's goal in clearer terms. View the paragraphs on page 3's end.

2. **On comment 2:** The grammatical errors in the paper need to be looked at. There are so many subject-verb agreement errors and inconsistencies in some of the sentences. I advised earlier for the service of a proofreader and I still think the author(s) need that.

**Answer:**

Greetings, reviewers We value the helpful criticism you have provided. The linguistic editing is done with great care.
